# Autism spectrum disorders and neuropathology of the cerebellum

**DOI:** 10.3389/fnins.2015.00420

**Published:** 2015-11-06

**Authors:** David R. Hampson, Gene J. Blatt

**Affiliations:** ^1^Leslie Dan Faculty of Pharmacy, Department of Pharmaceutical Sciences, University of TorontoToronto, ON, Canada; ^2^Program in Neuroscience, Hussman Institute for AutismBaltimore, MD, USA

**Keywords:** apraxia of speech, cerebellar cognitive affective syndrome, cerebellar vermis, deep cerebellar nuclei, fragile X syndrome, inferior olivary complex, Purkinje cell, tuberous sclerosis

## Abstract

The cerebellum contains the largest number of neurons and synapses of any structure in the central nervous system. The concept that the cerebellum is solely involved in fine motor function has become outdated; substantial evidence has accumulated linking the cerebellum with higher cognitive functions including language. Cerebellar deficits have been implicated in autism for more than two decades. The computational power of the cerebellum is essential for many, if not most of the processes that are perturbed in autism including language and communication, social interactions, stereotyped behavior, motor activity and motor coordination, and higher cognitive functions. The link between autism and cerebellar dysfunction should not be surprising to those who study its cellular, physiological, and functional properties. Postmortem studies have revealed neuropathological abnormalities in cerebellar cellular architecture while studies on mouse lines with cell loss or mutations in single genes restricted to cerebellar Purkinje cells have also strongly implicated this brain structure in contributing to the autistic phenotype. This connection has been further substantiated by studies investigating brain damage in humans restricted to the cerebellum. In this review, we summarize advances in research on idiopathic autism and three genetic forms of autism that highlight the key roles that the cerebellum plays in this spectrum of neurodevelopmental disorders.

## Autism spectrum disorders: clinical features, diagnostic criteria, classification, and etiology

Autism spectrum disorder (ASD, also known as autism spectrum condition), is a behaviorally defined neurodevelopmental disorder that is estimated to affect 1 in 88 children and is diagnosed approximately four times more frequently in males than in females (Halladay et al., 2015). Two international psychiatric classification systems are used for making clinical diagnoses: the Diagnostic and Statistical Manual of Mental Disorders (DSM) and the International Classification of Diseases. The diagnostic criteria for ASD are outlined in the widely used DSM where key revisions were put in place when DSM-IV was replaced by DSM-5 in 2013. The DSM-IV diagnostic criteria for Autistic Disorder defined three domains of behavior—social deficits, communication deficits, and repetitive/stereotypic behaviors. Under DSM-5 only two behavioral domains, social communication deficits and repetitive/stereotypic behaviors are stipulated (see Lai et al., 2013; Fung and Hardan, 2014 for reviews on this topic). Additional salient changes in DSM-5 include the elimination of both Asperger's Syndrome and Pervasive Developmental Disorder as diagnostic categories, while a new category, Social Communication Disorder, has been added. Regarding the latter, persons displaying impaired social communication but not engaging in repetitive behaviors (or restricted interests) could now be diagnosed with Social Communication Disorder (Fung and Hardan, 2014).

The level of cognitive impairment in ASD varies over a wide range. In a study conducted on 156 ASD patients between ages of 10 and 14, 55% had intellectual disability (IQ < 70), 17% had below average intelligence (IQ 70–84), 25% had average intelligence (IQ 85–114), and 3% had above average intelligence (IQ > 115) (Charman, 2011). However, it is important to note that standard intelligence tests of IQ are not optimal for assessing much of the autism population, due in part to difficulties in language and communication (Baum et al., 2014). In addition to cognitive impairment and the core symptoms, there are several additional conditions associated with ASD that are correlated with each other and vary greatly in their severity. These include seizures, motor impairments, altered sleep, and increased anxiety (Matson et al., 2007; Maski et al., 2011). Many persons with ASD also experience heightened or reduced sensitivity to sensory stimuli such as sound and temperature.

Approximately 90% of autism cases are classified as idiopathic while about 10% are caused by known gene mutations (Betancur, 2011; Zafeiriou et al., 2013). The genetic forms of ASD are widely studied in humans and animal models in part because knowledge obtained may prove valuable for enhancing our understanding of idiopathic autism. Examples of ASDs caused by gene mutations include fragile X syndrome (FXS) and tuberous sclerosis (TSC) which have mutations in the FMR1 and TSC1/2 genes, respectively. Rett syndrome was previously classified under autism spectrum disorders but it has not been retained under the ASD umbrella in DSM 5. However, clinically, Rett syndrome patients frequently show autism spectrum characteristics and as such, Rett can serve as a model system for ASD. Although FXS and TSC are two of the most common genetic causes of autism, individual genes linked to ASD are each responsible for only a minor proportion of autism cases; other genetic forms of ASD account for even fewer cases of autism, typically 1% or less. However, the extent of epigenetic effects and regulatory changes also needs to be considered and remains under investigation. Estimates of the incidence of FXS and TSC in the overall ASD population vary considerably. In an analysis of patient records from almost 15,000 persons with ASD, Kohane et al. (2012) reported an incidence of 0.5% for FXS and 0.8% for TSC. Others have reported higher incidences of FXS ranging up to about 5% of the ASD population (Budimirovic and Kaufmann, 2011). It has been estimated that about 30% of patients with FXS and about 50–60% with TSC present with ASD core symptoms (Kong et al., 2014). However, in the case of FXS, Budimirovic and Kaufmann (2011) have stated that “the high frequency of relatively mild autistic features, and differences in ascertainment strategies and supportive diagnostic methods have led to reported rates of autism within the FXS population that range widely from 15 to 60% for prevalence of ASD in males with FXS.” Therefore, relatively common but mild autistic characteristics can complicate the diagnosis of ASD in FXS.

Although much effort has centered on the identification of genes linked to ASD, there is also a growing trend toward more emphasis on investigating possible environmental causes. Examples include altered immune responses (Mead and Ashwood, 2015), maternal immune activation where a strong immune stimulus or infection during pregnancy is associated with an increased probability of giving birth to a child with ASD (Atladóttir et al., 2010a,b; Brown, 2012; Knuesel et al., 2014), and environmental contaminants (Kalkbrenner et al., 2014; Nevison, 2014; Posar and Visconti, 2014). However, these links so far are not well-established and will require more intense investigation to validate.

## Structure and anatomy of the cerebellum

Recent work has suggested that the mammalian cerebellum has undergone substantial enlargement throughout the evolution of apes including humans, and that its size has increased much more than predicted based on analogous enlargement of the cerebral cortex. As a result, humans and other apes deviated significantly from the general evolutionary trend for neocortex and cerebellum to change in tandem, leading to a significantly larger cerebella relative to neocortex size than other primates (Weaver, 2005). In fact, it has even been suggested that “given the role of the cerebellum in sensory-motor control and in learning complex action sequences, cerebellar specialization is likely to have underpinned the evolution of humans' advanced technological capacities, which in turn may have been a preadaptation for language” (Barton and Venditti, 2014). The large expansion of the cerebellar hemispheres has been particularly prominent in the posterior lobe of the cerebellum, a substructure that is crucial for rapid processing of cognitive and language skills (Broussard, 2014).

**Figure 1:**
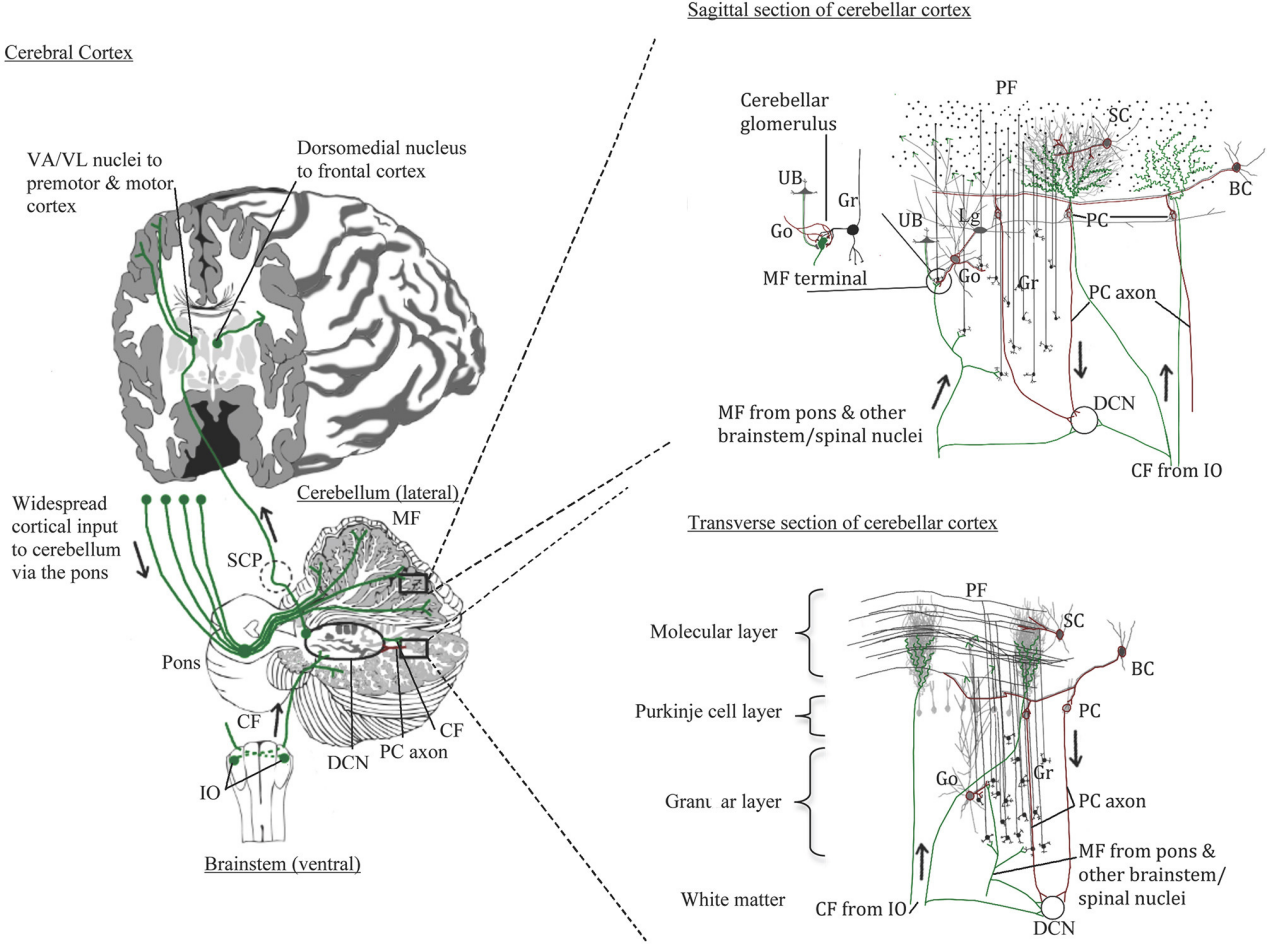
**Normal cerebellar circuitry**. Main inputs to the cerebellum are from the inferior olivary nuclei via the inferior cerebellar peduncle providing climbing fibers (CF) to Purkinje cells (PC) and, from various spinal cord and brainstem nuclei including the pons that relays cerebral cortical input through the middle cerebellar peduncle to granule cells (Gr). Both climbing and mossy fibers (MF) send collaterals to cells in the deep cerebellar nuclei (DCN). Granule cells in turn send their parallel fibers to PC dendrites in the molecular layer. The PCs innervated by climbing fibers send their axons to the same DCN cells that climbing fibers innervate. Excitatory DCN cells project to the thalamus and cerebral cortex; inhibitory DCN cells project back to the inferior olive completing the loops. Other cerebellar neurons include the Golgi cells (Go) that send inhibitory input to the mossy fiber glomeruli and has dendrites extending into the lower molecular layer where it contacts parallel fibers; Lugaro cells (Lg) that contact multiple PCs via its horizontal dendrites thus sensing the local environment and also contacts Go cells; and unipolar brush cells (UB) found mostly in cerebellar areas with vestibular functions and sends its axon to the Gr cell glomeruli, similar to Go cells.

The cerebellum comprises only about 10% of the brain's volume yet contains over half of all the neurons in the brain (Herculano-Houzel, 2010). In humans, the cerebellar cortex expanded with the cerebral cortex, especially in the hemispheric region (Balsters et al., 2010). As illustrated in Figure 1, the cerebellar cortex has one of the simplest configurations in the human brain. It is comprised of three layers: inner granular layer (GL), Purkinje cell layer (PCL), and outer molecular layer (ML). The underlying white matter contains the deep cerebellar nuclei, the main output region of the cerebellum. In development, billions of excitatory granule cells (GCs) have descended from the pia surface via the external granule layer to take residence in the GL, leaving “T-shaped” axons (i.e., parallel fibers, PF) that run parallel to the long axis of the cerebellar folia like telephone wires, synapsing on the elaborate dendritic arbors of GABAergic Purkinje cells (PCs) and continuing across the ML for long distances (e.g., Altman and Bayer, 1978). Each PC receives up to 100,000 PF synapses, and PC axons mainly travel to one of four pairs of deep cerebellar nuclei (fastigial, emboliform, globose, and dentate nuclei), but also give off collaterals to Lugaro cells. Lugaro cells, lie just beneath the PCL and contain long horizontal dendrites that can contact up to 10–15 PCs, hypothesized to monitor the environment around PCs (Lainé and Axelrad, 1998). Lugaro cell axons contact cerebellar interneurons in the ML and large inhibitory Golgi cells in the GL (Lainé and Axelrad, 1998). The intrinsic GABAergic interneurons in the ML include basket cells in the lower third of the ML that form perisomatic nests around PC somata and, stellate cells that reside in the outer two thirds of the ML and innervate PC dendrites. The large inhibitory Golgi cells in the GL, characterized physiologically by Eccles et al. (1967) and first called large stellate cells by Cajal, synapse on GC glomeruli and are part of an inhibitory feedback loop. Golgi cells also synapse on excitatory local circuit neurons (Mugnaini et al., 2011), the unipolar brush cells. Unipolar brush cells, initially described by Altman and Bayer (1978), have a round nucleus and a short tuft of dendrioles with large synaptic junctions with filapodia, which are thought to be involved in cell signaling. In the cerebellar cortex, unipolar brush cells are most abundant in regions involved in vestibular functions such as the vermis, flocculus, and paraflocculus (Diño et al., 2001; Englund et al., 2006). Granule cell axons (PFs) also form synaptic contacts on basket and Golgi cells, the latter of which, via its inhibition on GCs, can counteract the excitatory inputs onto PCs (Brodal, 1969).

A major input to the cerebellar cortex is excitatory (glutamate, aspartate) olivocerebellar climbing fibers (CFs) that cross at the level of the inferior olive, travel through the inferior cerebellar peduncle and the cerebellar white matter, and “climb” onto the primary, secondary and tertiary branches of PC arbors. In development, CFs innervate the PC soma but as the PC dendrites arborize, these connections are replaced by basket cell perisomatic “baskets.” Each PC receives only one CF and in the human CFs can innervate up to 10 PCs along its ascent (Chan-Palay, 1977). CFs give off collaterals to the deep cerebellar neurons on the same neurons that receives its PC innervation. Climbing fibers are thus topographically organized in parasagittal innervation patterns, but are less organized in patches laterally (Groenewegen et al., 1979). Inferior olivary neurons are electrotonically coupled and fire synchronously in groups thus activating clusters of PCs. Climbing fibers, which carry information from spinal cord and select brainstem nuclei as well as sensory and motor cortices generate complex spike excitatory postsynaptic potentials that are important in regulating the timing of PC activity, especially for motor function contributing to the “error correction system” of cerebellar activity (Eccles, 1967). More recently, cerebellar function in cognitive tasks has been recognized, and CFs may play an important role in contributing to a wide range of high order sensory associative cognitive behaviors.

Cerebellar mossy fibers produce simple spikes and arise from a variety of pre-cerebellar nuclei. Vestibulocerebellar fibers originate from vestibular ganglia and vestibular nuclei; spinocerebellar fibers arise from the dorsal spinocerebellar tract via the Clark's column (nucleus dorsalis); additional afferents arise in the anterior (ventral) spinocerebellar tract, cuneocerebellar tract (from the external cuneate nucleus), trigeminocerebellar fibers, lateral reticular fibers, and others. A massive projection arises from the cerebral cortex and travels through the pons (the cerebro-ponto-cerebellar tract) via the middle cerebellar peduncle. The topography of the cerebro-ponto-cerebellar projection has been well-established from tract tracing studies in primates (Schmahmann and Pandya, 1989, 1991, 1995, 1997a,b). Mossy fibers send collaterals to the respective deep cerebellar nuclei with the main axon terminating on GC dendrite glomeruli along with axon terminals from inhibitory Golgi cells and in some regions, unipolar brush cells. Thus, there is abundant sensori-motor input to the cerebellum from widespread regions of the cerebral cortex and the topographical ordering in the pons is relayed throughout region-specific areas of the cerebellum. For example, frontal projections via select parts of the pons project to the lateral hemisphere (Schmahmann and Pandya, 1995), mostly to the Crus I and II regions which have been implicated in high order cognitive functions (Schmahmann, 2010).

In terms of efferent projections, PC axons distribute topographically to the four deep cerebellar nuclei which in turn project through the crossed superior cerebellar peduncle mainly to the ventral anterior and ventral lateral nuclei of the thalamus and on to premotor and motor cortex. Other deep cerebellar nuclei projections include the red nucleus which gives rise to the rubrospinal tract, the vestibular nuclei (vestibulospinal tract), and other brainstem nuclei. Chan-Palay (1977) described GABAergic projection neurons in the deep cerebellar nuclei in rats to the inferior olivary complex that are different from intrinsic smaller GABAergic interneurons in the deep cerebellar nuclei (also articulated in Llinas et al., 2004). Some PC axons project directly to vestibular nuclei bypassing the deep cerebellar nuclei. Strick and colleagues used trans-synaptic transport via tracer injections in the frontal cortex and found strong labeling in Crus I and II regions via the mediodorsal thalamic nucleus (Strick et al., 2009). These studies demonstrated that the cerebellar hemisphere not only receives frontal lobe inputs, but also has a means to send projections back to these high-order association areas of the frontal lobe.

## Links between ASD and the cerebellum—information gleaned from assessing damage to the human cerebellum

What aspects of ASD could be most affected by cerebellar pathology? There have been several excellent reviews on this topic in recent years (e.g., Fatemi et al., 2012; Reeber et al., 2013; Rogers et al., 2013; Wang et al., 2014; Ebrahimi-Fakhari and Sahin, 2015); our aim here is to update and extend the discussion on this key issue in ASD research. Although the cerebellum has previously been associated primarily with motor functions, it is increasingly accepted that it is also involved in cognitive functions, due in part to its interconnections with other brain regions including (via the thalamus) the cerebral cortex (Fatemi et al., 2012; Broussard, 2014). Because most of the known pathological brain alterations in ASD are not restricted solely to the cerebellum but extend to other brain regions (see below), an examination of cases of cerebellar congenital abnormalities, injury (e.g., trauma, stroke), and disease (e.g., tumors) is instructive. Collectively, the cognitive and affective symptoms have suggested that lesions in specific site(s) in the adult cerebellum produce effects throughout connected networks in the brain, simulating functional deficits from thalamic and/or cerebral cortical lesions that lie upstream within the circuits. Schmahmann postulated that the cerebellum is an important regulator of the speed, consistency and appropriateness of cognitive processes, and that patients with cerebellar damage were unable to control their thought processes (Schmahmann, 2004). This led Ito (2008) to develop his “internal-model hypothesis for mental activities” that forms an analogy between the cerebellums' role of regulating the control of motor function with its modulation of mental activities. The model involves the concerted activity of large scale encoded neural circuits including the prefrontal cortex, temporoparietal cortex, and the cerebellar hemispheres, each of which participate in some aspect of the temporally sequenced network activity.

Although the descriptions of the effects of cerebellar lesions are extremely variable, useful information gleaned from descriptions of human lesions of the cerebellum indicates that both the location of the lesion and the age at which the damage occurred are critical factors in determining the outcome and any potential recovery (Strick et al., 2009; Manto, 2010). As delineated further below, in addition to fine motor error correction, the cerebellum also plays a critical role in visuospatial perception, auditory processing, verbal memory, sequencing, executive functions, and language (Bolduc et al., 2011, 2012; Broussard, 2014). To illustrate one example, auditory and written language comprehension necessitates attention to the message of interest—and suppression of interference from distracting sources. In a scan of multiple brain regions of typically developing individuals using structural and fMRI imaging, Filippi et al. (2011) showed that the only brain region of significance was the right posterior paravermis which showed a strong correlation between gray matter density and the control of verbal interference.

Autism is intimately associated with impaired communication. One manifestation of this is the difficulty with speech. Although DSM-5 does not incorporate specific criteria for deficits in speech and verbal communication, poorly integrated verbal and non-verbal communication is an example under the criteria for non-verbal communicative deficits. Additionally, individuals who meet criteria for autism spectrum disorder can also be further described with the specifier “with or without accompanying language impairment” (Fung and Hardan, 2014). Pertinent to this issue are recent findings that demonstrate an association between children with ASD and childhood apraxia of speech. Childhood apraxia of speech is defined as a neurological childhood speech sound disorder in which the precision and consistency of movements underlying speech are impaired in the absence of neuromuscular deficits. Children with apraxia of speech cannot produce the sounds necessary for speech due to deficits in motor planning, organization, and production. The muscles required for speech production are fully functional, but the ability to coordinate and plan motor movements to produce fluid intelligible speech is impaired. It is important to note that analysis of apraxia of speech can be difficult to disentangle from the clinical phenotype of ASD. Nevertheless, in a recent study, Tierney et al. (2015) examined young children with autism over the course of 3 years; 64% of children initially diagnosed with autism were found to also have apraxia of speech, and 37% of children initially diagnosed with apraxia also had autism (Tierney et al., 2015). The subjects with apraxia had “difficulty coordinating the use of their tongue, lips, mouth, and jaw and with accurately producing speech sounds, so that each time they say the same word, it comes out differently, and even their parents have difficulty understanding them.” This study also showed that the commonly used Checklist for Autism Spectrum Disorder accurately diagnoses autism in children with apraxia.

In the context of this review, one critical question is, how important is the cerebellum in verbal communication? The cerebellum is often, perhaps arbitrarily, perceived as being responsible for automatic movements and reflexes but not higher functions such as speech. However, that distinction may be meaningless. In fact speech requires a great deal of coordination that is largely automatic. As noted by Broussard (2014) “some apraxias such as gait apraxia and limb-kinetic apraxia are routinely associated with cerebellar damage but speech apraxia can also result from cerebellar damage.” Early documentation of this was described in a one-of-a-kind study of World War I soldiers with gunshot injuries restricted to the cerebellum where (Holmes, 1917) reported that speech was impaired in many of the casualties he examined. In another classic paper Schmahmann and Sherman (1998), reported on 20 cases where neurological disease was confined to the cerebellum. This group coined the term “cerebellar cognitive affective syndrome” to describe this patient population. Affected individuals exhibited impaired executive functions such as planning, set shifting, abstract reasoning, working memory, and difficulties with spatial cognition. Prominent features of this syndrome are language deficits and impaired verbal fluency (Schmahmann and Sherman, 1998). Some of these patients also presented with changes in their personalities including passivity, flattening, or blunting of emotion, decreased emotional expression and disinhibited or child-like behavior, often due to lesions in the vermis (see below and also Schmahmann, 2000, 2004, 2010).

**Figure 2:**
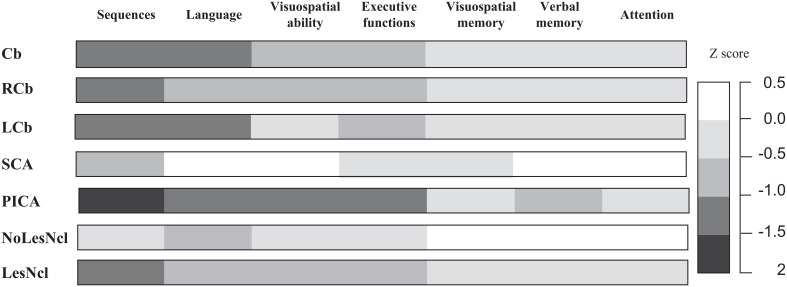
**Cognitive patterns of subjects with focal cerebellar lesions grouped according to lesion topography**. To compare performances between tests, raw scores were converted to *Z*-scores (*z* score = subject raw score minus population mean)/population standard deviation. For each cognitive function, a single *Z*-score was obtained by calculating the mean *Z*-scores of the tests, grouped according to that function. *Z* scores of −1 or lower were considered to indicate pathology. Cb, cerebellar damage; LCb, left cerebellar damage; LesNcl, lesions of the deep cerebellar nuclei; NoLesNcl, no lesions of the deep cerebellar nuclei; PICA, posterior inferior cerebellar artery; RCb, right cerebellar damage; SCA, superior cerebellar artery. Adapted with permission from Tedesco et al. (2011).

The findings of Schmahmann and Sherman (1998) have been validated in a study by Tedesco et al. (2011) who retrospectively analysed patient records from the Ataxia Lab of Santa Lucia Foundation in Italy. Of the 223 medical charts reviewed, 156 were included in the study which focused on the role of the cerebellum in cognition and the relevance of lesion topography in defining the cognitive domains that were affected. Vascular topography and the involvement of deep cerebellar nuclei were established as the chief factors that determined the cognitive profile. Language, executive function, visuospatial abilities and sequences were the most adversely affected functions irrespective of whether the damage was focal or degenerative. Differences in the patterns appeared when the location of the focal lesion was considered. The vascular territory of the superior cerebellar artery primarily involves the anterior lobe, whereas that of the posterior inferior cerebellar artery mostly involves the posterior lobe including the inferior aspects of the cerebellar hemispheres and vermis, and also the dentate nucleus. Strokes in the superior cerebellar artery or posterior inferior cerebellar artery usually present with gait problems and/or ataxia depending on which aspects of the arteries are blocked (e.g., ataxia of stance and gait are a sign of medial superior cerebellar artery and lateral posterior inferior cerebellar artery infarction; Timmann et al., 2009). Strokes in the anterior inferior cerebellar artery which supplies the flocculus, and inferior and anterior aspects of the cerebellum and middle cerebellar peduncles, are rare and usually affect the brainstem. Therefore, assessment of patients with stroke lesions in the superior cerebellar artery or posterior inferior cerebellar artery territory allowed evaluation of anterior vs. posterior cerebellar lesions. Intriguingly, subjects with posterior inferior cerebellar artery strokes performed worse than patients with superior cerebellar artery on all cognitive domains, and in fact, statistically significant differences were noted with regard to verbal memory, language, visuospatial and executive functions (see Figure 2).

Another general feature that has emerged from recent studies is that injury or malformations of the vermis result in disabilities that resemble those encountered in ASD. In the context of mouse models of ASD, support for an essential role of the vermis is illustrated by GABA_*A*_ receptor β3 (GABRB3) subunit knockout mice, which display autistic behaviors and selective vermal hypoplasia (DeLorey et al., 2008). The GABRB3^−∕−^ mouse has significantly decreased sagittal surface area of lobules II-VII measured semi-quantitatively and exhibits a reduced level of interaction with unfamiliar mice compared to controls. Specifically, GABRB3^−∕−^ mice show reduced social engagement in both sociability (interaction time with novel mouse) and social novelty testing (adding a second novel mouse, i.e., unfamiliar mouse to a chamber on the opposite side as the first now familiar mouse). In addition, the GABRB3^−∕−^ mouse in the DeLorey et al. (2008) study, exhibited hyperactivity and spent more time in the open portion of a circle than with the novel object in an exploratory behavior task and a stereotypical circling pattern. Although these authors mention that the vermal aplasia could contribute to the reported behavioral deficits in the GABRB3^−∕−^ mice, a previous study by Pierce and Courchesne (2001) did significantly correlate the degree of hypoplasia of vermal lobules VI-VII in children with ASD with reduced visuospatial exploration and with stereotyped behavior. In the context of human ASD, genetic studies have suggested that the GABRB3 gene may be a susceptibility locus (see Chen et al., 2014 and references therein) and duplication of a segment of chromosome 15q (“duplication 15 syndrome”), which contains several genes including the GABRB3 gene, occurs in approximately 2% of autism cases (Scoles et al., 2011). Moreover, postmortem samples from a small number of subjects have indicated reduced GABRB3 transcripts (Scoles et al., 2011) and protein (Fatemi and Folsom, 2011).

The vermis is phylogenetically the most ancient structure of the cerebellum (the paleocerebellum) and develops and becomes fully foliated by 4 months gestation in humans, while development of the large cerebellar hemispheres (neocerebellum) lags behind that of the vermis by 1–2 months. Studies conducted on children that have undergone surgery for cerebellar tumors affecting the vermis have also been informative. Typical problems include cognitive impairment and flattened affect manifesting as increased irritability, impulsiveness, disinhibition, poor attention and behavioral modulation (Levisohn et al., 2000; Riva and Giorgi, 2000). In an important study by Tavano et al. (2007), the clinical picture of 27 patients with congenital malformations restricted to the cerebellum was described in detail. Seventy-four percent of these patients presented with some degree of mental retardation. Notably, patients with cerebellar vermal agenesis or diffuse cerebellar hypoplasia presented with core ASD symptoms including language deficits, social interaction impairments, and some repetitive and stereotyped behaviors. In contrast, in cases where the lesions were restricted to the cerebellar hemispheres, the disabilities were less severe. Interestingly, motor deficits in patients with lesions in the vermis were less severe than patients with lesions in the cerebellar hemispheres. Overall, abnormalities affecting the vermis translated into the least favorable long-term outcomes and the most severe impairments, including pervasive developmental disorder, mental retardation, impaired social and communicative behavior, and social withdrawal.

In adults, damage to the cerebellar vermis may be relatively rare because some of these patients likely do not survive. However, a condition known as Dandy-Walker malformation primarily affects the vermis. The pathology consists of cerebellar vermal hypoplasia with upward vermis rotation and often elevation of the torcula, an enlarged fourth ventricle which extends posteriorly as a retrocerebellar cyst, and hydrocephalus which is present in 50–80% of subjects. This condition often presents with macrocephaly in the neonatal period, and infants may come to medical attention because of hydrocephalus, developmental delay, or ataxia (Parisi and Dobyns, 2003). Apnea and seizures are seen in a significant proportion of children and about one quarter of patients with this condition die. No studies have specifically investigated the incidence of ASD in Dandy-Walker malformations. However, developmental delay and mental retardation are common but highly variable in Dandy-Walker; as noted by Parisi and Dobyns (2003), the distribution of intelligence scores appears to be bimodal, suggesting that there may be two distinct groups: those with normal cognition (47%), and those with severe impairment (IQ < 55), which represented 35% of the cohort.

## Cerebellar pathology in ASD—human postmortem and imaging studies

### Idiopathic autism

Early neuropathological studies examining eight serially sectioned autism brains revealed a reduction in PCs most pronounced in the lateral hemispheric region coupled with a pallor of Nissl staining in the granular cell layer in the same region (Bauman and Kemper, 1985; Kemper and Bauman, 1993). The reduction in PC density was later reported by a number of investigators (e.g., Bailey et al., 1998; Whitney et al., 2008; Skefos et al., 2014) and in total, about 75% of reported autism cases that involved quantitative studies had a reduction in PCs (see Schumann and Nordahl, 2011 for review). In contrast, the medial vermis displayed a less severe reduction in PCs (Bauman and Kemper, 1985 and others). Fatemi et al. (2002a) reported a 24% decrease in the size of remaining PCs in the cerebellum in autism cases compared to controls.

An open question is, with the reduction in PCs in the autism cerebellum, how is the distribution of CFs affected? An insight into this question was found in studies of spontaneous cerebellar mutants that despite having decreases in PCs, the CFs distributed in a topographically normal pattern (Blatt and Eisenman, 1988, 1989, 1993). Electrophysiological studies in the adult homozygous weaver mouse revealed that CFs hyperinnervated the PCs in the vermis, with each PC receiving up to four CF inputs (Crepel and Mariani, 1976; Puro and Woodward, 1977; Mariani, 1982) instead of the normal monoinnervation first described anatomically by Ramon y Cajal in the late nineteenth century. It remains to be determined whether the autism brain has a similar PC multiple innervation pattern due to decreased PCs. This may contribute to sustain inferior olivary neurons, which are unaffected in number in the medulla in postmortem autism cases (Blatt, 2012).

**Figure 3:**
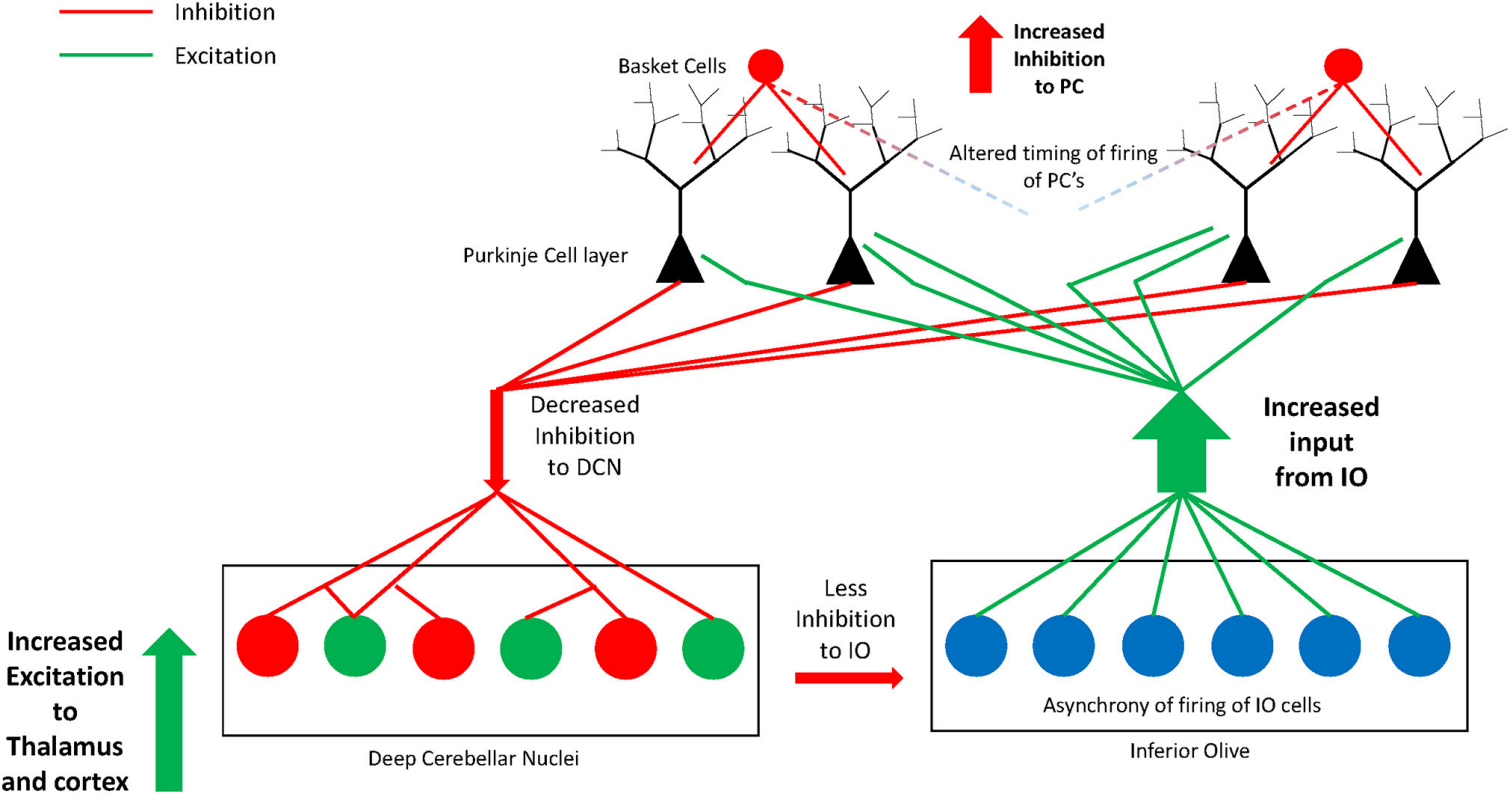
**Illustration of the hypothesis of increased excitatory:inhibitory circuitry within the cerebellum in autism**. Missing Purkinje cells may disrupt olivocerebellar circuitry in individuals with autism. Some of the remaining Purkinje cells are likely multi-innervated by climbing fibers as previously shown in animal models of spontaneous cerebellar mutations. Remaining Purkinje cells have decreased GAD67 mRNA and protein that could translate to decreased GABA inhibition to excitatory cells in the deep cerebellar nuclei. The deep cerebellar nuclear cells in turn send increased excitatory input to thalamus and cerebral cortex thus affecting higher circuitry within the network. The circuitry may be further perturbed by an abnormal nucleo-olivary projection from the deep nuclei to the inferior olive. GABAergic projection cells in the deep nuclei (dentate nucleus) have decreased GAD65 mRNA and project to the inferior olive. This can result in asynchrony of firing of groups of inferior olivary neurons whose altered climbing fiber complex spike activity would cause abnormal timing of firing in select subgroups of Purkinje cells. Thus, alterations in cerebellar circuitry in autism likely contribute to motor and non-motor aspects of the phenotype by affecting higher-order association cortices within the networks.

We hypothesize that due to the absence of some PCs, those that border remaining clusters are likely to be multi-innervated by excitatory olivocerebellar CFs (illustrated in Figure 3). The remaining PCs in the cerebellar hemisphere in postmortem cases contain decreased GAD67 mRNA (Yip et al., 2007) suggesting abnormal functional connectivity with the deep cerebellar nuclei. In the autism cerebellum, there was low Nissl staining in serial sections through the deep cerebellar nuclei in Bauman and Kemper's (1985), cases suggesting a possible deficiency in cell number and/or density in this key output structure. In that same study and confirmed by Blatt (2012), the inferior olivary complex contained ectopic linearly-oriented neurons in the principal olive on the lateral edge of the ribbon, that may disrupt the electrotonically coupled synchrony of firing of principal olivary neurons and affect the timing of PC activity (Welsh, 2002; Welsh et al., 2005). There were also age-related changes in the size of inferior olivary neurons with larger cells in children with autism and smaller neurons in adults (Bauman and Kemper, 1985). Interestingly, the principal olive has a robust projection to the lateral cerebellar hemisphere, the cerebellar region that contains the greatest decrease in PCs (Bauman and Kemper, 1985; Whitney et al., 2008; Skefos et al., 2014), and is the recipient of cortical association inputs especially from the frontal lobe via the pons (Schmahmann and Pandya, 1995, 1997a; see Ramnani, 2012 for review). Neurochemical studies in the Crus II region revealed decreased glutamic acid decarboxylase 67 (GAD67) mRNA in the remaining PCs in young adults with ASD (Yip et al., 2007), and subsequent studies showed likely compensatory increases in GAD67 mRNA in cerebellar interneurons in the molecular layer (Yip et al., 2008).

Fatemi et al. (2002b) demonstrated an overall reduction in GAD in the cerebellum using specimens from different cerebellar regions in autistic brains. Yip et al. (2009) added a study analyzing the GABAergic neurons within the dentate nuclei and found significantly decreased GAD65 mRNA levels in the adult autism group in a larger sized subpopulation of GABAergic neurons, described by Chan-Palay (1977) as projecting to inferior olivary neurons in rodents and confirmed by Llinas et al. (2004). In contrast, the smaller sized GABAergic interneurons contained normal GAD65 mRNA levels (Yip et al., 2009). If the anatomy is similar in primates, that is, if these larger GABA-containing neurons of the dentate do project to the inferior olive, then it could potentially have a profound effect on the synchrony of firing of olivary neurons and influence the timing of the remaining PCs in the hemisphere (see Figure 3). These events could help reaffirm one of the more interesting hypotheses of disturbed neural circuitry in autism resulting in an excitatory:inhibitory imbalance (Blatt et al., 2001; Hussman, 2001; Rubenstein and Merzenich, 2003; Pacey et al., 2009; Blatt, 2012) affecting motor and non-motor functional activity.

Additional observations on postmortem autism brains include flocculonodular dysplasia and hypoplastic lobes, which may indicate cell migration abnormalities to defined areas (Wegiel et al., 2010). Cytoarchitectural laminar abnormalities are not common in autism but have been reported sporadically in the anterior cingulate (Simms et al., 2009), posterior cingulate (Oblak et al., 2011) and, the fusiform gyrus (van Kooten et al., 2008). Reports of neuroglia at pathologic sites in the autism brain were reported by Bailey et al. (1998) who found increased glial fibrillary acidic protein (GFAP) in Bergmann glia in the cerebellum in three cases of autism and increased glial proliferation in one case. Vargas et al. (2005) also reported increased Bergmann glia reactivity in the PC layer and increased microglia reactivity in the granular layer and accompanying white matter in 9 of 10 patients. These investigators also demonstrated activated microglia in the vicinity of degenerating PCs and granule cells (Vargas et al., 2005) but it is unclear whether epilepsy and/or anti-epileptic therapies contributed to the cell loss and subsequent increased glial activity.

### Antibodies to cerebellar antigens

Serum autoantibodies directed against human brain were identified via ELISA and Western immunoblot in children with ASD and their non-autistic siblings as denser bands of 73 kDa in the cerebellum and cingulate cortex and, as 100 kDa bands in the caudate, putamen, and prefrontal cortex compared to controls (Singer et al., 2006). Serum antibodies from mothers of children with autism have the capability to cross the placenta and possibly alter brain development (Zimmerman et al., 2007). The Van de Water group followed with a study extracting human cerebellar protein with a 52 kDa band present in about 20% of children with autism (Wills et al., 2009). This same cerebellar extract was examined via immunohistochemical staining of antibodies in Macaque fascicularis monkey cerebellum. Intense staining was found in Golgi cells and, in some cases, basket cells in the deep molecular layer. Furthermore, these antibodies also immunostained Golgi cells in 21% of children with ASD (7/34) compared to controls (0/23). No relationship was found between antibodies for the 52 kDa protein or with immunohistochemical staining, when compared against the behavioral outcome which was based on subjects with a diagnosis of regressive vs. early onset autism (Wills et al., 2009). Although unclear, autoantibodies may bind to and affect the physiological role of these key inhibitory neurons in the cerebellum with downstream pathological effects. In a later study, GABAergic interneurons were found to be co-localized with calcium binding proteins including calbindin, parvalbumin, and calretinin and were immunoreactive in additional brain regions (Wills et al., 2011). In another study, 45 and 62 kDa bands were isolated from Western blot of autoantibodies toward target cerebellar proteins (Goines et al., 2011). The presence of the 45 kDa protein autoantibodies were associated with the diagnosis of autism whereas the 62 kDa protein autoantibodies were associated with ASD diagnosis (Goines et al., 2011). However, children with autism, ASD and typically developing children that contain reactivity to these autoantibodies, all displayed altered basic cognitive and adaptive behavioral traits (Goines et al., 2011). Plasma samples from children with or without subtypes of ASD were immunostained against monkey cerebellum and other brain regions including dentate gyrus, were significantly correlated with behavioral and emotional problems utilizing the Child Behavior checklist (Rossi et al., 2011). A comprehensive study examining the behavioral profiles in Italian children with ASD and their unaffected siblings was reported by Piras et al. (2014). In that study, the 45 and 62 kDa cerebellar specific antibodies correlated with cognitive impairment and motor stereotypies, respectively, in the ASD group and both correlated with increased head circumference. Also, the presence of maternal anti-brain autoantibodies of 37, 39, and 73 kDa in children with ASD correlated with cognitive behavioral abnormalities including verbal and non-verbal language development, sleep/wake cycle alterations and developmental delay (Piras et al., 2014). The authors also found a correlation between the mothers of autistic children having the 39 or 73 kDa autoantibodies directed against the fetal brain, with the presence of the 62 kDa autoantibodies in the ASD child. Therefore, this is an exciting area of research that may represent an important subset of ASD children with neurologic profiles based on immunological factors including maternal contribution.

### Neuroimaging studies

A discussion of neuroimaging studies in ASD should be prefaced with the suggestion that there may be a disconnection between neuropathological reports from postmortem cases with moderate to severe forms of idiopathic autism, vs. imaging studies that are primarily conducted in higher functioning individuals. Very few high functioning or Asperger's autism postmortem cases have been obtained from the brain banks that would match up well with the higher functioning cohorts typically utilized in imaging studies. It therefore remains unclear whether alterations in brain activity within specific patients or cohorts are due to observed postmortem changes in histology of the same brain area(s).

Early neuroimaging studies of the cerebellum in ASD reported a reduction in the size of cerebellar regions including the vermis (Hashimoto et al., 1995; Kaufmann et al., 2003). Another study confirmed that the size of the vermis is indeed reduced in ASD but noted that the reduction in vermal volume did not correlate with symptom severity or with verbal, non-verbal or full scale IQ (Webb et al., 2009). In a pathology report, Kemper and Bauman (1998) found much variability of the size of different lobules in the vermis with widening of the spaces of the folia. These neuropathologic observations may explain the contrasting imaging results in the vermis in autism cases. For example, Courchesne et al. (1988) reported a significantly smaller mean area of vermal lobules VI and VII but not lobules I-V and reported hypoplasia (16% smaller) or hyperplasia (34% larger) of vermal lobules VI and VII across ages in a subgroup of autism patients (Courchesne et al., 1994). A smaller cerebellar vermis in autism cases was also reported by others (e.g., Kaufmann et al., 2003; Scott et al., 2009). In contrast, Piven et al. (1997) did not replicate these findings but did find that the total cerebellar volume was increased in ASD. However, the increase in cerebellar volume was found to be in general, and proportional to total brain volume.

A meta-analysis of structural MRI studies in the literature by Stanfield et al. (2008) reported the general findings of increased total brain volume and volume of the cerebral hemispheres, cerebellum and caudate, whereas in contrast, the corpus callosum volume was reduced. These authors also concluded that there was an age and IQ effect on the volume of vermal lobules VI-VII and for age in the amygdala. In contrast, a well-designed structural MRI study was conducted by Scott et al. (2009) that included low functioning individuals with ASD, high functioning individuals with autism, an Asperger's group, and a typically developing control group amongst 62 male subjects 7.5–18.5 years old. The authors concluded that the midsagittal area of the vermis or of subgroups of vermal lobules in any of the autism groups was not significantly different from typically developing children and neither age nor IQ predicted the size of the vermis in the autism groups (Scott et al., 2009). Other early observations included an increase in cerebellar white matter volume, and a reduction in the gray/white matter ratio (Courchesne and Pierce, 2005). In a study of 103 subjects, Cheung et al. (2009) using diffusion tensor imaging, reported a correlation of repetitive behaviors via ADI-C diagnostic algorithm scores with reduced cerebellar white matter fractional anisotropy indices. In another study, significant differences in white matter volume in the autism group included the corticoponto-cerebellar tract, arcuate fasciculus, uncinated fasciculus and corticospinal tract (Ecker et al., 2012).

An MRI study by Bolduc et al. (2012) on young children with developmental cerebellar pathologies found that the lateral cerebellar hemisphere is important in cognitive skills and language. These authors also found that there is a stronger association between language skills and the right cerebellar hemisphere, previously demonstrated in tumor resection studies (Levisohn et al., 2000; Riva and Giorgi, 2000). Using voxel-based morphometry, Ecker et al. (2012) found a negative correlation between cerebellar gray matter volume vs. group (ASD vs. controls). Similar findings were found in the posterior cingulate, fusiform gyrus (posterior), and amygdala. In contrast, using partial least squares analysis, in many regions in the autism brain, these authors concluded that there is positive correlation with gray matter volume in the following areas: the cerebellar tonsil, dorsomedial prefrontal cortex, fusiform gyrus (anterior) parahippocampal gyrus, superior temporal gyrus, mid-and anterior-cingulate, inferior frontal gyrus, caudate, and insular cortex.

## Syndromic forms of autism—postmortem, imaging, and mouse studies

### Fragile X syndrome

FXS is the most common form of X-linked intellectual learning disability affecting about 1 in 2500 individuals, and is associated with a cytogenic marker in the distal region of the long arm of the X chromosome. The disorder is caused by an unstable trinucleotide repeat of cytosine guanine guanine (CGG) near the 5′ promotor region of the FMR1 gene. Normal individuals have 5–55 CGG repeats whereas in FXS, repeat lengths of greater than 200 causes gene hypermethlyation and loss of the FMR1 gene product, Fragile X mental retardation protein (FMRP). FXS is associated with abnormal dendritic development and maturation of synaptic connectivity that contributes to the cognitive deficits (see Bagni et al., 2012; Gallagher and Hallahan, 2012 for reviews). Approximately 20% of individuals with FXS have epilepsy (Berry-Kravis, 2002).

FXS is associated with abnormalities in cortical dendritic spines; they are longer, thinner, and lack the mushroom-shaped spines that are characteristic of mature dendrites (Hinton et al., 1991; Irwin et al., 2000, 2001, 2002). As seen in idiopathic autism, cerebellar PC loss and cell displacement have been reported in a human postmortem study of FXS (Greco et al., 2011). Cerebellar pathology has also been noted in adult onset pre-mutation carriers of fragile X where repeat lengths are in the intermediate range (55–199). These carriers are susceptible to fragile X-associated tremor ataxia syndrome (FXTAS), a neurodegenerative condition that typically occurs in late middle age (Hagerman and Hagerman, 2015). Neuropathology in this syndrome is associated with patchy loss of axons in the brain, spongiosis of the middle cerebellar peduncles, and loss of PCs (Greco et al., 2002, 2006, see Gallagher and Hallahan, 2012, for review).

There are some data indicating that that the posterior-superior vermis is significantly larger in boys with FXS and autism than in boys with FXS without ASD; these changes appear to be specific to the individuals with ASD (i.e., autistic disorder) since boys with FXS and social anxiety show increased anterior, but not posterior vermis size (Kaufmann et al., 2003). Moreover, Gothelf et al. (2008) reported positive correlations between size of the posterior vermis (and the caudate) and several subscales of the Autism Behavior Checklist in persons with FXS. Curiously, the increased posterior-superior cerebellar vermis size affects the same region in FXS (i.e., lobules VI–VII) that is smaller in individuals with idiopathic ASD (Budimirovic and Kaufmann, 2011).

#### The fragile X knockout mouse

The Fmr1 knockout (KO) mouse is generally considered a good animal model of human FXS and has been intensively studied on the molecular, cellular, anatomical, and behavioral levels (Hampson et al., 2012). Results from studies on the Fmr1 mouse were instrumental in facilitating translation of metabotropic glutamate receptor 5 antagonists and a GABA_B_ receptor agonist into human clinical trials, although those conducted to date have all failed due to lack of efficacy and/or meeting endpoints. Nevertheless, this mutant mouse continues to provide useful information on the neurobiology of FXS, and will likely continue to be used for preclinical drug studies.

In the context of neuroanatomical aberrations, a recent study classified the Fmr1 mouse in relation to other mouse models of ASD. Most studies investigating mutant ASD mice have examined only a single mouse model. However, Ellegood et al. (2015) pursued a more holistic approach whereby the clustering of autism was studied based on anatomical phenotyping (using various modes of high resolution MRI) of 27 different ASD mouse lines. The results indicated that ASD mouse lines separated into three major clusters; a cerebellar set, a cortico-thalamic-striatal set, and a set related to limbic structures (Ellegood et al., 2015). The strongest connections in the clustering of models were between En2, Nrxn1α, and Fmr1. This cluster (Group 1), which also included Shank 3 mice, displayed increases in large white matter structures including the corpus callosum, fimbria, and fornix, as well as increases in the frontal and parieto-temporal lobe, and decreases in the cerebellar cortex. It is intriguing that the Fmr1 mouse appears to be anatomically related to the En2 knockout mouse as the En2 gene is a homeobox gene that is essential for the proper development of the cerebellum (Cheng et al., 2010; Choi et al., 2014). Previous analyses on the Fmr1 knockout mouse conducted by this group provided insight into how the cerebellum is affected by the loss of FMRP. Ellegood et al. (2010) used high resolution MRI imaging to show that of 62 brain regions examined, the cerebellum stood out as one of the few regions showing significant volume changes compared to wild-type controls. Specifically, it was demonstrated that the deep cerebellar nuclei, which as noted above, transmit the sole output of the cerebellar cortex to the thalamus and cerebral cortex, were smaller in volume in the Fmr1 mice. Moreover, immunocytochemical analyses indicated a loss of neurons and an increase in astrocytes in the deep cerebellar nuclei (Ellegood et al., 2010). Pacey et al. (2013) conducted a detailed study of postnatal development of the Fmr1 mouse cerebellum and showed reduced volume in the first few weeks after birth (as determined by diffusion tensor imaging), and a delay in myelination which was likely due to a reduced number of oligodendrocyte precursor cells. A delay in myelination may in turn affect the trajectory of neuronal maturation which could have permanent consequences affecting the adult CNS (Pacey et al., 2013).

### Rett syndrome

Rett syndrome, first discovered in the late 1970s/early 1980s occurs in 1/10,000–1/23,000 girls with 90% of cases involving a mutation in the X-linked methyl-CpG binding protein 2 gene (MECP2). Rett syndrome is a progressive neurological condition that presents early with autistic features, but then progresses to severe neurological and cognitive dysfunction. Rett primarily affects females where beginning at 12–18 months of age they regress, losing speech, and motor skills and suffer from additional problems including mental retardation, respiratory impairment, and seizures (Armstrong, 1992, 2005). Rett differs from ASD in that patients with Rett syndrome have a reduced lifespan, although greater than 70% live beyond age 45 (Tarquinio et al., 2015). The leading cause of premature death is cardiorespiratory failure. Mutations in MECP2 can result in cessation and regression of development in early childhood, ataxia, stereotypies as well as severe cognitive and spastic motor abnormalities (Ropers and Hamel, 2005). Patients have reduced brain weight (not accounted for by age-related atrophy) directed more toward the cerebral hemispheres than the cerebellum (Armstrong, 2005). The Rett syndrome brain is characterized by widespread reduced dendritic branching in many areas in the frontal and motor cortices, hippocampus, thalamus, basal ganglia and amygdala (Bauman et al., 1995). Also, decreased size of cortical minicolumns in select frontal and superior temporal areas was reported in five Rett patients with mean age of 14.4 years (Casanova et al., 2003).

In contrast, the cerebellar literature for Rett syndrome is very sparse. One account of five patients by Oldfors et al. (1990) reports that all five had smaller brains compared to controls by 25% with the cerebellum decreased proportionately in size. One patient had a patchy loss of PCs with several folia having a complete loss and, the ML had cell loss and gliosis. Another patient had moderate generalized loss of PCs and thinner folia with gliosis. The other patients had milder effects but also focal loss of PCs, minor gliosis in the cerebellar cortical layers and a few of the patients were observed to have a loss of myelin in the white matter (Oldfors et al., 1990). In addition to mutation of the MeCP2 in Rett syndrome, MECP2 gene duplication is also associated with severe neurological consequences including mental retardation, hypotonia, recurrent infections, and interestingly, cerebellar degeneration (Reardon et al., 2010).

Mouse studies specifically focusing on the cerebellum in Rett syndrome are also relatively sparse. Steadman et al. (2014) used MRI imaging to compare three genetic ASD mouse models: Neuroligin-3 R451C knock-in, integrin β3 homozygous knockout, and MECP2 308-truncation mice, and focused on assessing morphological differences specific to the cerebellum. All three ASD lines showed significant volume changes in the cerebellum. Of note is the observation that the posterior vermis was morphologically altered in the two mouse lines with reported repetitive behaviors (MeCP2 and integrin β3 mice). An unexpected finding by Steadman et al. (2014) was that MECP2 mutant mice had cerebellar volume changes that increased in scope depending on the genotype; compared to age and sex matched wild-type mice MeCP2 heterozygous males had reduced cerebellar volumes while homozygous females had increased cerebellar volumes.

### Tuberous sclerosis complex (TSC)

In a recent analysis of the field (Sundberg and Sahin, 2015) indicated that approximately 50% of patients with TSC present with ASD and that preclinical and clinical investigations conducted to date indicate the cerebellum has a profound regulatory role during development of social communication and repetitive behaviors in TSC. TSC is an autosomal dominant condition that affects cerebral cortical development (the lesions are called tubers), and in a smaller cohort, the development of the cerebellum, where reduced volume has been reported (Weisenfeld et al., 2013). The pathogenesis may lead to developmental delays, epilepsy, and autism. Vaughn et al. (2013) retrospectively reviewed MRI images from 145 children with TSC with a mean age of 7.6 years old. These investigators found cerebellar tubers present in 35 of 145 patients (24%) while 6 of 35 patients had cerebellar atrophy. This incidence of cerebellar tubers was in agreement with Ertan et al. (2010) who reported 8 out 27 patients (30%) had tubers while only 1/27 showed atrophy. Tubers in some patients changed while others remained static; in those that changed, the pathogenesis may have been due TSC2 gene mutations resulting in disordered migration and/or accelerated astroglial apoptosis (Chu-Shore et al., 2009). Previously, Jurkiewicz et al. (2006) selected 12 patients with TSC (median age of 10.6 years old) and 8 of 12 had a mutation in the TSC2 gene, with 50% displaying cerebellar atrophy. Further analysis of the cerebellum in TSC was reported in an MRI and PET imaging study of 78 patients with TSC; 27% had cerebellar pathology that was positively correlated with the severity of autistic symptoms (Eluvathingal et al., 2006). In this study, within-group analyses of the cerebellar lesion group revealed that children with right-sided cerebellar lesions had higher social isolation and communicative and developmental disturbance compared with children with left-sided cerebellar lesions. The side of the cerebellar lesion was not related to adaptive behavior functioning. These findings provide additional empiric support for a role the cerebellum in autistic symptomatology. It was concluded that further investigation of the potential role of the right cerebellum in autism, particularly with regard to the dentatothalamofrontal circuit, is warranted.

Several exceptionally informative lines of mutant Tsc mice have been generated and studied in detail. FXS and TSC are both caused by mutations in genes that regulate protein synthesis in neurons and it has been hypothesized that excessive protein synthesis is one core patho-physiological mechanism of intellectual disability and autism. Using electrophysiological and biochemical assays of neuronal protein synthesis in the hippocampus of Tsc2 ± and Fmr1 knockout mice, Auerbach et al. (2011) demonstrated that synaptic dysfunction caused by these mutations actually falls at opposite ends of a physiological spectrum. Synaptic, biochemical and cognitive defects in these mutants are corrected by treatments that modulate metabotropic glutamate receptor 5 in opposite directions, and deficits in the mutants disappear when the mice are bred to carry both mutations. It was concluded that normal synaptic plasticity and cognition occur within an optimal range of metabotropic glutamate-receptor-mediated protein synthesis, and deviations in either direction can lead to shared behavioral impairments. In terms of treatment, these findings support the concept that antagonists of mGluR5 (including negative allosteric modulators) could be used in FXS, while mGluR5 agonists (or positive allosteric modulators) might be useful for treating TSC.

Reith et al. (2011) studied a mutant mouse line in which the Tsc2 gene was selectively deleted from PCs starting at postnatal day 6. A crossed line was generated (Way et al., 2009) that produced mice with one functional Tsc2 allele (Tsc2f/-;Cre); this line was examined because these mice model a TSC patient with one non-functioning TSC2 allele. Somatic cell loss of the remaining Tsc2floxed allele occurs primarily in PCs. The loss of Tsc2 caused a progressive increase in PC cell size and subsequent death from apoptosis. PC loss was predominantly cell type specific and associated with motor deficits. Immunohistochemical analysis showed that both endoplasmic reticulum and oxidative stress were increased in Tsc2-null PCs. The cell death and ER stress phenotypes were rescued by treatment with the mTORC1 inhibitor rapamycin. To assess whether the murine PC loss had a correlate to the human TSC, postmortem cerebellum samples from TSC patients were examined and found to have PC loss in half of the samples. These findings support a role for the TSC complex in PC survival by regulating endoplasmic reticulum and oxidative stress and reveal a salient feature of TSC neuropathology.

In a subsequent study, (Reith et al., 2013) conducted a series of behavioral tests to determine if Tsc2flox/-;Cre mice displayed autistic-like deficits. Tsc2f/-;Cre mice demonstrated increased repetitive behavior as assessed with marble burying activity. Using the three chambered apparatus to assess social behavior, Tsc2f/-;Cre mice showed behavioral deficits by exhibiting no preference between a stranger mouse and an inanimate object, or between a novel and a familiar mouse. The observations indicate that selective loss of Tsc2 in PCs in a Tsc2-haploinsufficient background leads to autistic-like behavioral deficits.

Another mouse model of TSC has been described where the selective loss of Tsc1 gene expression only in PCs also resulted in autistic-like behaviors including abnormal social interaction, repetitive behavior and vocalizations (Tsai et al., 2012). In addition, PC loss was apparent in Tsc1 −/− mice at 2 months of age but not in +/− mice at 2 or 4 months of age. Most startling were the observations that (a) both Tsc1 −/− and +/− mice showed significant reductions in PC excitability (including reduced PC firing rates), and (b) that not only homozygous Tsc −/− mice, but also heterozygous Tsc1 +/− mice showed autistic like behaviors *in the absence or prior to any PC loss.* Importantly, the findings of Tsai et al. (2012) in Tsc1 +/− mice indicate that an autistic behavioral phenotype can be induced not only by the loss of PC, but also solely by impaired PC excitability.

In summary, mice with loss of Tsc1 or Tsc2 restricted to PCs both resulted in PC degeneration (in Tsc1 −/− mice and in Tsc2 +/− mice) and haploinsufficiency of either Tsc1 or Tsc2 is necessary and sufficient to induce an autistic phenotype in mice. These observations also overlap and are consistent with results from chimeric Lurcher mice which exhibit partial depletion of PCs during development and have been reported to display ASD characteristics such as motor hyperactivity, increased repetitive behavior, and deficits on a reversal learning task (Dickson et al., 2010; Martin et al., 2010). Collectively, these findings strongly indicate that PC loss and/or dysfunction may be an important link between TSC and ASD, and suggest the possibility that this phenomenon could contribute to other forms of ASD.

## Conclusions and open questions

We present the following major points gleaned from this review:

- Studies investigating congenital and acquired damage limited to the cerebellum generally support the proposition that many of the symptoms of ASD have a cerebellar component. Some studies appear to indicate that the vermis and lateral hemisphere might be considered regions of special interest.
- The cerebellum is intimately linked to many if not most of the behavioral symptoms seen in ASD. These include disruption or impairments in motor activity and coordination, repetitive behaviors, sensory perception, cognitive ability, and language and speech generation and comprehension. The involvement of the cerebellum in this array of different symptoms is likely a result of the absolute necessity of its powerful and rapid computational power in processing sensory, motor, and cognitive information in the brain.
- Overall, human brain imaging studies that included the cerebellum have reported highly variable results in ASD, especially in the vermis which also varies in size and volume in typically developing individuals. Imaging studies on mutant ASD mouse lines have suggested that a subset have significant alterations of cerebellar size and/or structure (e.g., fragile X, En2, Nrxn1α, and Shank 3 mice).
- Human postmortem analyses are still limited by relatively small numbers of individual brains examined. Nevertheless, collectively these studies indicate a loss of PCs in a high percentage of cases with idiopathic autism. This finding is additionally supported by a more limited data set from published cases of FXS and TSC. The fact that PCs are the sole output of the cerebellar cortex, and that their axons project to the deep cerebellar nuclei which in turn project directly and indirectly to multiple forebrain regions, places this unique cell type in a strategic position in information processing and overall brain function.
- Neurochemical studies in postmortem autism cerebella have revealed alterations in GAD65/67 in PCs, basket cells and in the dentate nuclei suggesting an imbalance of excitatory:inhibitory circuitry within the lateral hemisphere Crus II region. Further, cytoarchitectural abnormalities in the inferior olivary complex and in its inhibitory modulation from the dentate may lead to asynchrony of firing of inferior olivary neurons altering excitatory input to the remaining PCs. PCs bordering areas of absent PCs are hypothesized to be multi-innervated, thus affecting cerebellar output to high order association cortices.
- Results from human imaging and neuropathological studies of TSC strongly support an important contribution of cerebellar pathology to the overall clinical picture including the autism spectrum characteristics associated with the disorder. Additionally, results from Tsc mice with mutations in Tsc1 and Tsc2 genes restricted only to PCs have been particularly enlightening. These studies complement human neuropathological findings demonstrating a reduction in PCs and provide strong support for an essential role of the cerebellum in ASD.
- The demonstration by Tsai et al. (2012) from the analysis of mice with PC-restricted mutations in Tsc1 showing that autistic symptoms occur in the presence of altered PC excitability, but prior to PC loss, is particularly intriguing. This finding suggests that impaired PC excitability might be sufficient to induce ASD symptoms, and that cases where PC loss can be demonstrated may represent a later stage pathological state.
- Future human and animal studies of ASD should incorporate, and perhaps give priority to, assessing cerebellar parameters at the molecular, cellular, anatomical, physiological, and behavioral levels.

The following questions arise delineating potential priorities for future studies to address:

- Could reduced numbers of PCs, or even intact but dysfunctional PCs, be a feature common to many cases or forms of ASD? If so, this would represent a clear focus for future efforts in ASD research. In addition to the recent novel findings from mutant Tsc mice, the issue of the status of PC excitability and other physiological properties of PCs should be explored in other models of syndromic ASDs.
- In light of PC loss in the autism cerebellum, how is the distribution of CFs affected? It remains to be determined whether the autism brain displays a PC multiple innervation pattern due to decreased PCs. This may contribute to sustain inferior olivary neurons, which are unaffected in number in the medulla in postmortem autism cases.
- What is the relationship between the cerebellum and the basal ganglia in autism? Both structures are now known to participate in motor and non-motor functions, have indirect connectivity via the subthalamic nucleus, and, have reciprocal connectivity either directly or indirectly with similar thalamic nuclei and cortical targets. Could the two brain regions both participate in the alteration of fine motor control and high order cognitive behaviors in autism?

### Conflict of interest statement

The authors declare that the research was conducted in the absence of any commercial or financial relationships that could be construed as a potential conflict of interest.
